# Age Differences and Prevalence of Comorbidities for Death and Survival in Patients with COVID-19: A Single-Center Observational Study in a Region of Southern Italy

**DOI:** 10.3390/life14111376

**Published:** 2024-10-25

**Authors:** Biagio Santella, Silvana Mirella Aliberti, Luigi Fortino, Antonio Donato, Vincenzo Andretta, Emanuela Santoro, Gianluigi Franci, Mario Capunzo, Giovanni Boccia

**Affiliations:** 1 Department of Medicine, Surgery and Dentistry “Scuola Medica Salernitana”, University of Salerno, 84081 Salerno, Italy; bsantella@unisa.it (B.S.); sialiberti@unisa.it (S.M.A.); lfortino@unisa.it (L.F.); adonato@unisa.it (A.D.); vandretta@unisa.it (V.A.); esantoro@unisa.it (E.S.); gfranci@unisa.it (G.F.); mcapunzo@unisa.it (M.C.); 2 DAI Department of Health Hygiene and Evaluative Medicine, A.O.U. San Giovanni di Dio e Ruggi d’Aragona, 84131 Salerno, Italy

**Keywords:** age group, comorbidity, chronic diseases, COVID-19, public health, SARS-CoV-2, survival, mortality

## Abstract

The SARS-CoV-2 outbreak has resulted in a considerable number of deaths worldwide. The virus damages the pulmonary artery endothelium, leading to a condition known as microvascular pulmonary inflammatory thrombotic syndrome (MPITS), which can be fatal and cause multiple organ failure. The presence of preexisting comorbidities has been shown to significantly impact the severity and prognosis of patients with SARS-CoV-2 infection. The objective of this study was to compare the age groups of patients with coronavirus disease 2019 (COVID-19) and to identify the prevalence of comorbidities associated with death and survival in an area of southern Italy. The data set consisted of 1985 patients with confirmed cases of SARS-CoV-2 infection who were admitted to the A.O.U. San Giovanni di Dio e Ruggi d’Aragona Hospital in Salerno between January 2021 and December 2022. The results were presented for the overall population and stratified by outcome and age group. All analyses were performed using the XLSTAT (Lumivero, 2024, Paris, France) and STATA software (release 16.1, StataCorp LLG, College Station, TX, USA, 2019) packages. In the study, population, 636 cases (32%) resulted in death, with a higher prevalence in the 60–79 age group, followed by the ≥80 and 30–59 age groups. The most prevalent diseases among deceased and surviving patients with confirmed cases of SARS-CoV-2 infection were those affecting the circulatory system (61.5% vs. 55.5%), the respiratory system (55.8% vs. 26.2%), and the metabolic system (25.9% vs. 25.4%). In patients aged 30–79, respiratory diseases were the primary cause of mortality, whereas in those aged ≥80, circulatory system diseases were more prevalent. Among survivors, cardiovascular diseases were the most common comorbidities across all age groups, followed by respiratory diseases and endocrine, metabolic, and immune disorders. Moreover, these comorbidities were associated with an elevated risk of mortality. The study emphasizes the substantial influence of age and comorbidities on the mortality associated with SARS-CoV-2 infection. These findings highlight the necessity for targeted interventions to manage comorbid conditions in patients with SARS-CoV-2 infection, particularly in older adults.

## 1. Introduction

Since the declaration of the public health emergency (PHE) due to the novel coronavirus (2019-nCoV) in January 2020 [1], all nations around the world have taken a series of unprecedented actions to support public health [2,3,4]. These measures have had a considerable and unparalleled influence on the entirety of the health ecosystem, facilitating the acceleration of diagnostic and immunization initiatives, and enhancing coverage and accessibility to healthcare resources.

Three years after the declaration of the PHE due to the novel coronavirus, the latest data from Johns Hopkins University [5], collected through 10 March 2023, indicated that the virus has caused more than 6.8 million deaths globally.

A variety of factors contributed to the mortality and severity of SARS-CoV-2 infection. Chronic noncommunicable diseases, comorbidities, and coinfections were particularly important [6]. Patients diagnosed with COVID-19 who also had hypertension, obesity, chronic lung disease, diabetes, or cardiovascular disease had an increased risk of developing severe outcomes, including pneumonia and acute respiratory distress syndrome (ARDS) [6,7,8]. Individuals with chronic kidney disease, cancer, and old patients admitted to long-term care facilities had a significantly increased risk of contracting the SARS-CoV-2 virus and succumbing to its effects [9]. Preexisting comorbidities (especially diabetes and overweight) also increased the risk of vaccine breakthrough infection. Furthermore, the SARS-CoV-2 virus has been identified as the causative agent in microvascular pulmonary inflammatory thrombotic syndrome and pulmonary artery endothelial damage. As a result, the thrombotic manifestations lead to obstructed normal blood flow, and the disease has the potential to spread from the lungs to other vital organ systems (e.g., the heart, brain, and kidneys). Ultimately, this can lead to the deterioration of multiple organs and, in some cases, death [10]. In individuals who test positive for SARS-CoV-2, an increased risk of disease severity has been associated with the presence of preexisting endothelial dysfunction, a condition commonly associated with diabetes and cardiovascular disease [11]. The study by Grippo et al. [12] confirmed that the most common comorbidities associated with SARS-CoV-2 were hypertension, diabetes, ischemic heart disease, and obesity. In contrast, Vetrano et al. [13] showed that the presence of multiple cardiorespiratory, metabolic, and neuropsychiatric disorders was more common than expected in patients who died from SARS-CoV-2 infection in Italian hospitals.

Fauci et al. [14] pointed out that risk factors for the development of SARS-CoV-2 infection and the likelihood of mortality in adults include a number of demographic characteristics, including older age, male gender, and ethnicity, as well as the presence of underlying medical conditions such as cardiovascular disease, hypertension, and chronic obstructive pulmonary disease (COPD). Similarly, younger people and those with specific comorbidities, such as obesity, have been reported to be at a heightened risk for infection and potentially more severe outcomes from SARS-CoV-2 infection [15,16,17,18,19].

In addition to the aforementioned factors, the severity and mortality associated with SARS-CoV-2 infection were also influenced by laboratory indices such as pro-inflammatory cytokine levels and complications [20,21,22,23], as well as the potential influence of SARS-CoV-2 in the early stages of megakaryocyte by increasing platelet production and activation [24]. Furthermore, there has been increasing interest in the impact of lifestyle and environmental factors, including smoking, vitamin D deficiency, and air pollution, play in the development of obesity [25,26,27,28,29].

It has been established that age and comorbidities represent the most significant risk factors for major morbidity and mortality in patients with coronavirus disease 2019 (COVID-19) due to senescence-related events and deleterious inflammation [30]. This conclusion is based on evidence from a multitude of sources worldwide. The case fatality rate (CFR) of SARS-CoV-2 infection increased with age, as shown in studies by Wu et al. [31] and Zhu et al. [32]. Conversely, in Italy, the mortality rate decreased in the oldest old population [33]. In accordance with this, it has been shown that long-lived individuals (LLI) have elevated circulating levels of BPIFB4, a protein implicated in the homeostatic response to inflammatory stimuli. This makes LLI less susceptible to inflammation [30] and more resistant to SARS-CoV-2 infection [30,34]. Furthermore, the study by Verduri et al. [35] revealed a reduced mortality rate among the older population between the initial (2020) and subsequent (2021) waves of the SARS-CoV-2 pandemic. The second wave cohort exhibited a notable reduction in frailty and disease severity. Conversely, the meta-analysis conducted by Pranata et al. [36] showed that a high clinical frailty score was associated with an increased risk of mortality in old patients with SARS-CoV-2 infection. Other studies have supported the idea that the aging process is accompanied by a state of chronic low-grade inflammation, termed “inflammaging” [37], which can lead to the development of frailty [38] and predispose to the onset of SARS-CoV-2 infection [39,40]. The majority of those who have succumbed to complications from the novel Coronavirus disease (2019-nCoV), otherwise known as SARS-CoV-2, have been affected by multimorbidity, defined as the co-occurrence of two or more chronic medical conditions in the same individual [41]. This phenomenon has been observed in multiple case series and reports. In a prior report concerning the pre-infection health status of deceased individuals in Italy, it was found that approximately 84% of these individuals had a history of multimorbidity [13]. Among these individuals, the most prevalent chronic diseases were ischemic heart disease and atrial fibrillation. It has been confirmed by numerous studies that those with preexisting chronic diseases are at increased risk of adverse outcomes when infected with SARS-CoV-2 [13,41,42]. It has also been well established that individuals with multiple chronic diseases were more likely to have a higher number of comorbidities than would be expected from the case alone. This phenomenon could be attributed to a number of factors, including the presence of common risk factors and a similar underlying pathophysiology [43]. In addition, the development of severe COVID-19 was a risk factor for the persistence of symptoms after the acute illness. In fact, it was expected that patients recovering from severe respiratory illness might experience a variable recovery period, depending on several factors, in particular, the virulence of the pathogen and individual susceptibility [44,45].

In consideration of the aforementioned factors, this study aims to compare patients with diagnosed cases of SARS-CoV-2 infection across different age groups, with the objective of identifying the prevalence of comorbidities associated with mortality and survival in a region of southern Italy. An understanding of these correlations is essential for the development of targeted interventions and the improvement of patient outcomes in future pandemics.

## 2. Materials and Methods

### 2.1. Study Design and Data Collection Procedure

This study employs a single-center observational design to determine the prevalence of comorbidities associated with mortality and survival in patients belonging to designated age-specific cohorts. Patients were divided into three age groups (30–59, 60–79, and ≥80 years) to represent distinct life stages, each with varying risk profiles for COVID-19 severity. This categorization follows common epidemiological practices, allowing for a comparison of mortality rates and comorbidities across adults, old and oldest, who exhibit distinct clinical characteristics.

The data were collected from January 2021 to December 2022, based on a report from the A.O.U. San Giovanni di Dio e Ruggi D’Aragona University Hospital in Salerno, Campania, Italy. The data set included all patients who had been confirmed positive for SARS-CoV-2 via nucleic acid real-time polymerase chain reaction (RT-PCR) testing of throat swabs. Preexisting respiratory diseases, such as chronic lung conditions, were analyzed separately from COVID-19 infection, which was considered an aggravating factor for patients with these conditions, potentially influencing outcomes (mortality or survival) based on disease severity.

Hospital reports were obtained from designated institutions, with strict measures in place to ensure confidentiality. Patients’ data were anonymized, with only age group identifiers visible. In addition to data on patients infected with SARS-CoV-2, the reports included information on concomitant diseases categorized according to the International Statistical Classification of Diseases and Related Health Problems (ICD-10-CM), as well as data on patient survival or death during hospitalization.

### 2.2. Ethical Consideration Statement

The study was conducted according to the guidelines of the Declaration of Helsinki. Approval for this study was obtained from the authority of A.O.U. San Giovanni di Dio e Ruggi d’Aragona Hospital. This is a retrospective study; the data collected were anonymous and not linked to any sensitive patient information, only age and comorbidities status.

### 2.3. Statistical Analysis

The personal and clinical characteristics of patients infected with SARS-CoV-2 were described using summary statistics. Descriptive frequency analyses were expressed in absolute numbers and percentages. Patients were classified into three age categories, with the first category beginning at 30 years of age. A chi-squared test was used to determine whether there were any significant differences between the variables of deceased and surviving patients. Results were reported for the whole population and stratified by outcome and age group. Multivariate Poisson regression was also used to determine the influence of different comorbidity categories on COVID-19 mortality age differences. The analysis also examined the incidence ratio of all age groups on mortality, as well as the effect of the number of comorbidities on mortality outcomes. Logistic regression was used to examine the relationship between the total number of comorbidities and patients’ survival. An alpha level of 0.05 was considered statistically significant. All analyses were conducted using XLSTAT (Lumivero, 2024, Paris, France) and STATA software (release 16.1, StataCorp LLG, College Station, TX, USA, 2019).

## 3. Results

### 3.1. Characteristics of the Whole Sample

**Figure 1:**
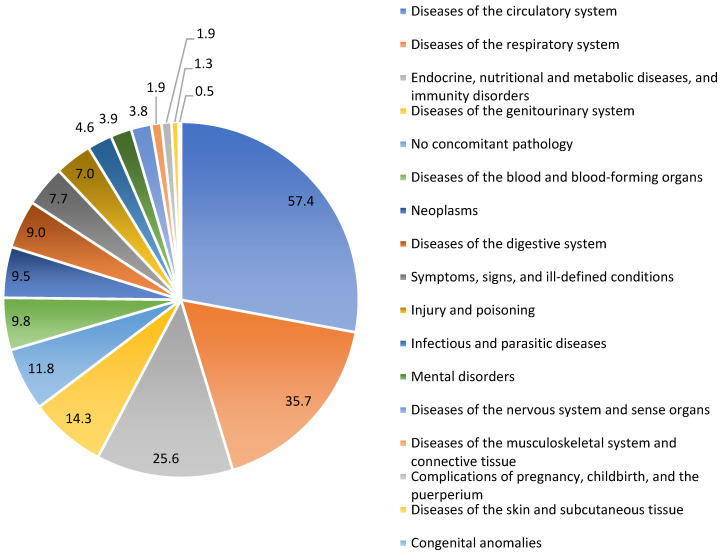
The proportion of comorbidities among the total number of patients included in the study.

A total of 1985 patients with confirmed cases of SARS-CoV-2 infection were included in this study, with ages ranging from 30 to over 90 years. At the time of hospitalization, the majority of patients had underlying chronic noncommunicable diseases, including but not limited to diseases of the circulatory system, respiratory system, endocrine glands, nutrition, metabolism, immune disorders, and the genitourinary system (Figure 1).

Of the total number of patients, 636 (32%) died, while 1349 (68%) survived. The most prevalent diseases among the deceased and survived patients with confirmed cases of SARS-CoV-2 infection were those affecting the circulatory system (61.5% vs. 55.5%), the respiratory system (55.8% vs. 26.2%), and the metabolic system (25.9% vs. 25.4%) (Table 1).

### 3.2. Differences in Mortality and Survival Rates Between Age Groups in Relation to Comorbidity

**Figure 2:**
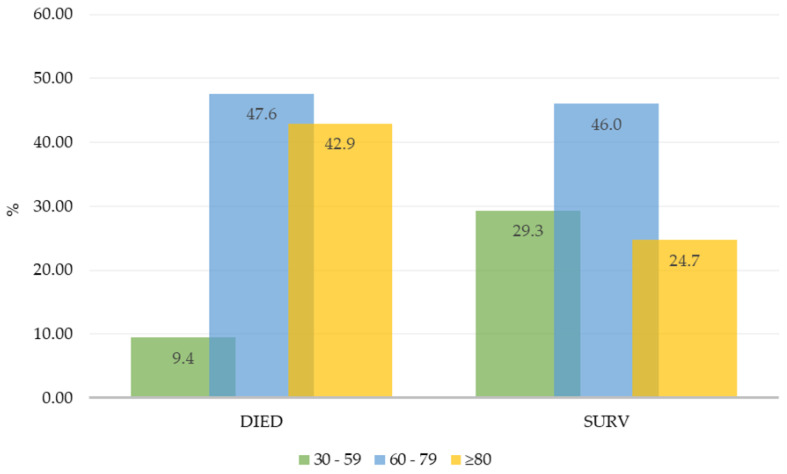
The group of deaths and survivors in each age group among hospitalized patients.

A total of 1985 hospitalized patients with confirmed cases of SARS-CoV-2 infection, 1349 survived and 636 died, were categorized into three age groups: 30–59 years (adults), 60–79 years (old), and 80+ years (oldest). Among these, the 60–79 age group had the highest mortality rate, followed by the 80+ age group, while the 30–59 age group had the lowest mortality rate (Figure 2).

In the 60–79 age group, respiratory diseases accounted for 60.4% of deaths, circulatory diseases for 56.8%, and endocrine, nutritional, metabolic, and immune disorders for 26.1%. Notably, 11.2% of these cases had no concomitant pathology, while 10.9% had genitourinary diseases (Table 2). In comparison, the 30–59 age group showed a significantly lower proportion of deaths (Figure 2). Circulatory system diseases were the most commonly reported comorbidities among both deceased and surviving individuals across all age groups (Table 1). In the 30–59 age group, 55% of deceased and 33.7% of survivors had these conditions (X^2^ = 10.22; *p* = 0.002). Similarly, in the 60–79 age group, circulatory diseases were present in 56.8% of the deceased and 60.1% of the survivors (X^2^ = 0.91; *p* = 0.338). Among those aged ≥80, 68.1% of the deceased and 73% of survivors had circulatory disease (X^2^ = 1.70; *p* = 0.192). Notably, the survival rate for those with circulatory diseases was higher in the ≥80 group compared to younger groups. Another significant finding was the high prevalence of respiratory diseases, particularly in the 30–59 age group, where 80% of deaths were related to these conditions (X^2^ = 109.59; *p* < 0.001). Interestingly, individuals in the ≥80 group had the most favorable outcomes compared to younger groups, with a death rate of 45.4% and survival rate of 33.6% (X^2^ = 8.76; *p* = 0.003). Additionally, conditions related to the endocrine glands, nutrition, metabolism, and immune system varied significantly across age groups (Table 2), highlighting the differential impact of these comorbidities on different populations.

**Figure 3:**
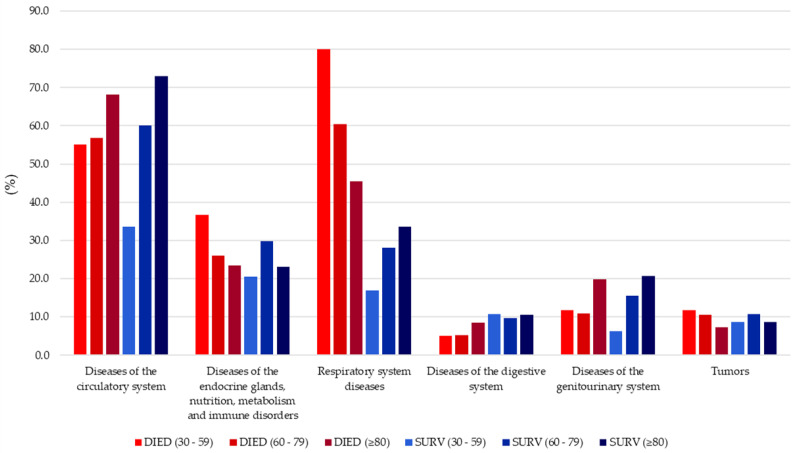
Analysis of comorbidities across age groups and their effect on mortality and survival in SARS-CoV-2 infected patients. For a comprehensive overview of comorbidities, refer to Table 2. It provides the complete data you need to gain valuable insights.

As illustrated in Figure 3, individuals aged 80 years and older with SARS-CoV-2 infection appear to demonstrate greater resilience to circulatory, respiratory, and genitourinary system disorders than the other two age groups.

The multivariate Poisson regression model constructed to examine the association between COVID-19 mortality age differences and the incidence of preexisting comorbidity showed that fifteen variables were statistically associated with the outcome (Model 1, Table 3). These included diseases of the circulatory system (IRR = 4.87; 95% CI 4.23–5.60; *p* < 0.001), diseases of the respiratory system (IRR = 3.02; 95% CI 2.60–3.50; *p* < 0.001), endocrine, nutritional, and metabolic disorders (IRR = 2.17; 95% CI 1.85–2.53; *p* < 0.001), and diseases of the genitourinary system (IRR = 1.21; 95% CI 1.02–1.44; *p* = 0.028) had a significant negative correlation. In contrast, neoplasms; diseases of the digestive system; symptoms, signs, and ill-defined conditions; injury and poisoning; infectious and parasitic diseases; mental disorders; diseases of the nervous system and sense organs; diseases of the musculoskeletal system and connective tissue; complications of pregnancy, childbirth and puerperium; diseases of the skin and subcutaneous tissue; and congenital anomalies were significantly positively associated with the outcome (Model 1 Table 3). Furthermore, the results of the multivariate Poisson regression analysis indicated that all three age groups were statistically significant predictors of mortality. The findings showed that the mortality incidence rate was significantly lower in the 30–59 age group (IRR = 0.85; 95% CI 0.77–0.95; *p* = 0.005) and in those ≥80 years of age (IRR = 0.82; 95% CI 0.76–0.88; *p* < 0.001). In contrast, the 60–79 age group exhibited an increased incidence risk ratio (IRR = 1.37; 95% CI 1.28–1.47; *p* < 0.001) (Model 2 Table 3). The logistic regression results indicate that comorbidities had a statistically significant impact on mortality (OR = 1.36; 95% CI = 1.01–1.81; *p* = 0.038) (Model 3 Table 3). This finding was further supported by the results of the multiple Poisson regression analysis, which demonstrated that the risk of a fatal outcome increased proportionally with the number of comorbidities (Model 4 Table 3).

## 4. Discussion

The objective of this study was to gain a more profound comprehension of the diverse chronic diseases and age groups of patients diagnosed with SARS-CoV-2. This was carried out with the intention of identifying the prevalence of comorbidities associated with mortality and survival in patients hospitalized in a region of southern Italy.

The present study demonstrated that patients with confirmed SARS-CoV-2 infection and preexisting cardiovascular, pulmonary, and metabolic comorbidities exhibited significant differences in incidence between survivors and non-survivors. Our regression results further elucidate the clinical significance of these characteristics. Chronic diseases were identified as a major risk factor for mortality, particularly in patients with severe symptoms requiring hospitalization, as these individuals were more likely to experience organ failure and, ultimately, fatal outcomes. The logistic regression analysis confirmed that comorbidities had a statistically significant impact on mortality, a finding that aligns with the work of Hasan et al. [46]. Their study similarly found that preexisting cardiovascular conditions, such as atrial fibrillation, heart failure, ischemic heart disease, and stroke, are associated with higher mortality in SARS-CoV-2 patients due to impaired hemodynamic responses to the virus. Moreover, the multiple Poisson regression analysis further reinforced these results, demonstrating that the risk of fatal outcomes increases proportionally with the number of comorbidities. The accumulation of multiple underlying conditions significantly elevated mortality risk across the patient population. Interestingly, while the presence of comorbidities increased the mortality risk, age also played a pivotal role. Our findings suggest that comorbidities, including cardiovascular, pulmonary, and metabolic complications, are associated with an increased risk of mortality, particularly in the 60–79 age group. This highlights the need for targeted clinical strategies based on both age and burden of comorbidities to improve patient outcomes.

It is plausible that the precipitous surge in cases of COVID-19 is attributable to a confluence of factors, including an inherent deficiency in cardiorespiratory fitness and compromised respiratory mechanics [47]. In our study, respiratory system complications were the most common cause of mortality. This finding is consistent with the observations of Agodi et al. [48], who reported that patients who succumbed to SARS-CoV-2 infection often exhibited comorbidities such as hypertensive heart disease, ischemic heart disease, cerebrovascular disease, diabetes mellitus, and chronic lower respiratory tract disease. It is noteworthy that the survival rate is higher in the age group of Oldest than in the other groups mentioned above for this chronic noncommunicable disease, as stated in our study. This finding is in agreement with the conclusion of the Istituto Superiore di Sanità [33] that the mortality rate among the oldest population has shown a decline. Indeed, long-lived individuals (LLIs) [34,49,50,51] have been demonstrated to exhibit resilience and to possess elevated circulating levels of BPIFB4, a protein implicated in the homeostatic response to inflammatory stimuli. This renders LLIs less susceptible to inflammatory processes and more resistant to SARS-CoV-2 infection [30].

Notable shifts were observed in the clinical manifestations of the disease in adults. The present study found that the number of deaths associated with COVID-19 in adult groups was lower than in other age groups, a finding that is consistent with that of other researchers in the field. It appears that mortality may be associated with the occurrence of complications, in contrast to what was observed in the old, where mortality was more closely linked to preexisting vulnerability due to the presence of chronic diseases. The data from 79,394 patients in China with confirmed cases of SARS-CoV-2 infections suggested that the mortality rate for patients aged 59 years and above may be approximately 5.1 times higher than that of patients aged 30–59 years after the onset of symptoms [52]. A meta-analysis involving 212 studies and 281,461 individuals from 11 countries/regions revealed that, among patients with severe COVID-19, the average age was 60.4 years, with more than half (61%) being male [53]. Another meta-analysis also indicated that patients aged >70 years may be at a higher risk of developing severe disease, requiring intensive care, and ultimately dying from the virus [54]. These findings align with our study, which observed that the 60–79 age group had the highest mortality rates, particularly in individuals with underlying health conditions such as circulatory, respiratory, and metabolic diseases.

Individuals with a history of cardiovascular disease were found to be at a greater risk of developing severe cases of the disease, given that the virus is capable of promoting acute cardiac injury [55]. This finding is in accordance with the results of our study, which indicated that diseases of the circulatory system were the most prevalent comorbidities among the patients included in the research. Conversely, numerous studies have demonstrated that the prevalence of comorbidities is higher among patients diagnosed with severe cases of COVID-19, encompassing a diverse array of conditions. These include cardiovascular disorders such as myocardial infarction and heart failure [56,57,58], hypertension [57], diabetes mellitus [56,57,58], chronic obstructive pulmonary disease (COPD) [57,58], neoplastic diseases [58,59], cerebrovascular pathologies [57], and chronic kidney diseases [60]. A meta-analysis revealed that comorbidities of hypertension, diabetes, and cardiovascular disease are associated with an elevated risk of COVID-19, intensive care unit (ICU) admission, and fatal outcomes across all age groups [61]. As postulated by Wolff and colleagues [23], SARS-CoV-2 is capable of damaging multiple organs, including the liver, kidney, heart, and others. The presence of preexisting comorbidities in these organs serves to further exacerbate the progression of SARS-CoV-2 infection, ultimately resulting in severe and fatal outcomes.

A notable limitation of this study is the absence of data on patients’ gender and detailed information regarding vaccination status. Gender is a well-established factor influencing both the severity and mortality rates of SARS-CoV-2 infection, with males generally experiencing more severe disease progression and higher mortality compared to females [62,63,64,65]. The lack of gender-specific data may introduce bias, limiting our ability to fully analyze gender-related differences in survival rates and comorbidities. Additionally, missing data on vaccination status—both of which significantly impact immunity and mortality—represent another potential confounder. Although the “Istituto Superiore di Sanità Report of 21 December 2021”, indicates high vaccination coverage in Italy—86.6% for the 30–59 age group, 92.2% for those aged 60–79, and 95.7% for individuals over 80 [66]—it remains unclear how many of the study participants were vaccinated. This gap in vaccination data could obscure its influence on survival rates, further complicating the analysis of the relationship between age, comorbidities, and outcomes.

Notwithstanding these constraints, this study provides valuable insights into the prevalence of preexisting morbidities among patients diagnosed with SARS-CoV-2, particularly in relation to age-specific outcomes. Our findings highlight the pivotal role of comorbidities in shaping the course of infection, underscoring a critical area that warrants further exploration. Future research should aim to incorporate comprehensive data on both gender and vaccination status to more accurately identify the factors influencing survival and mortality in COVID-19 patients. Doing so will help minimize the risk of bias and confounding, ultimately leading to more precise conclusions about the determinants of patient outcomes.

## 5. Conclusions

In summary, the study found a significant prevalence of preexisting comorbidities, particularly cardiovascular, pulmonary, and metabolic complications, in patients with confirmed SARS-CoV-2 infection. Major contributors to mortality included circulatory, respiratory, and endocrine-metabolic complications. Interestingly, survival rates were higher in patients over 80 years of age, especially those with circulatory, respiratory, and genitourinary disorders, while the 60–79 age group probably had the highest number of comorbidities, and this is somehow our expectation. These findings highlight the critical role of preexisting comorbidities in determining the outcomes of SARS-CoV-2 infection. Therefore, targeted management strategies for high-risk groups, particularly those with chronic health conditions, are essential to reduce mortality and improve survival. The study also highlights the need for further research to better understand these relationships and to develop tailored care protocols that address the specific needs of different age groups and comorbidity profiles.

## Figures and Tables

**Table 1:** The prevalence of comorbidities in patients who died and those who survived.

Comorbidity in Dead and Survived Patients	Died n = 636	Survived n = 1349	*p*
Diseases of the circulatory system	61.5%	55.5%	0.0123
Diseases of the respiratory system	55.8%	26.2%	<0.0001
Endocrine, nutritional and metabolic diseases, and immunity disorders	25.9%	25.4%	0.8054
Diseases of the genitourinary system	14.8%	14.1%	0.6797
No concomitant pathology	10.7%	12.3%	0.2982
Diseases of the blood and blood-forming organs	5.5%	11.8%	0.0001
Neoplasms	9.3%	9.6%	0.7987
Diseases of the digestive system	6.6%	10.1%	0.0099
Symptoms, signs, and ill-defined conditions	11.2%	6.0%	0.0001
Injury and poisoning	6.8%	7.0%	0.8182
Infectious and parasitic diseases	5.5%	4.2%	0.2064
Mental disorders	5.2%	3.3%	0.0474
Diseases of the nervous system and sense organs	3.8%	3.9%	0.9939
Diseases of the musculoskeletal system and connective tissue	1.3%	2.2%	0.1428
Complications of pregnancy, childbirth, and the puerperium	0.2%	2.7%	0.0001
Diseases of the skin and subcutaneous tissue	2.0%	1.0%	0.0482
Congenital anomalies	0.3%	0.5%	0.5270

Note: Chi-Square test—*p* value.

**Table 2:** The prevalence of comorbidities among patients who died and survived, stratified by age groups.

Comorbidities in Deceased and Survived	30–59 Years				60–79 Years				≥80 Years			
	Died		Surv		Died		Surv		Died		Surv	
	n.	%	n.	%	n.	%	n.	%	n.	%	n.	%
Diseases of the circulatory system	33	55.0	133	33.7	172	56.8	373	60.1	186	68.1	243	73.0
Diseases of the respiratory system	48	80.0	67	17.0	183	60.4	174	28.0	124	45.4	112	33.6
Endocrine, nutritional, metabolic diseases, immunity disorders	22	36.7	81	20.5	79	26.1	185	29.8	64	23.4	77	23.1
Diseases of the genitourinary system	7	11.7	25	6.3	33	10.9	96	15.5	54	19.8	69	20.7
No concomitant pathology	1	1.7	76	19.2	34	11.2	67	10.8	33	12.1	23	6.9
Diseases of the blood and blood-forming organs	4	6.7	35	8.9	13	4.3	64	10.3	18	6.6	60	18.0
Neoplasms	7	11.7	34	8.6	32	10.6	67	10.8	20	7.3	29	8.7
Diseases of the digestive system	3	5.0	42	10.6	16	5.3	60	9.7	23	8.4	35	10.5
Symptoms, signs, and ill-defined conditions	8	13.3	17	4.3	31	10.2	42	6.8	32	1.7	22	6.6
Injury and poisoning	2	3.3	15	3.8	12	4.0	40	6.4	29	10.6	40	12.0
Infectious and parasitic diseases	5	8.3	22	5.6	18	5.9	22	3.5	12	4.4	13	3.9
Mental disorders	2	3.3	11	2.8	13	4.3	17	2.7	18	6.6	17	5.1
Diseases of the nervous system and sense organs	1	1.7	18	4.6	15	5.0	16	2.6	8	2.9	17	5.1
Diseases of the musculoskeletal system and connective tissue	2	3.3	9	2.3	3	1.0	11	1.8	3	1.1	10	3.0
Complications of pregnancy, childbirth, and the puerperium	0	0.0	36	9.1	1	0.3	0	0.0	0	0.0	0	0.0
Diseases of the skin and subcutaneous tissue	0	0.0	1	0.3	7	2.3	9	1.4	6	2.2	3	0.9
Congenital anomalies	1	1.7	1	0.3	0	0.0	5	0.8	1	0.4	1	0.3
Total patients	60		395		303		621		273		333	

Note: n—number of cases; %—percentage of cases; Surv—survived.

**Table 3:** Results of multivariate Poisson and logistic regression analyses for the outcomes of interest across multiple predictive variables.

Model 1. COVID-19 Mortality Age Differences by Comorbidity Log Likelihood = −56.88, x^2^ = 4379.24 (16 df), *p* < 0.001	IRR	95% CI	*p*
Incidence of Preexisting comorbidities			
No concomitant pathology	1 ^a^		
Diseases of the circulatory system	4.87	4.23–5.60	<0.001
Diseases of the respiratory system	3.02	2.60–3.50	<0.001
Endocrine, nutritional, metabolic diseases, immunity disorders	2.17	1.85–2.53	<0.001
Diseases of the genitourinary system	1.21	1.02–1.44	0.028
Diseases of the blood and blood-forming organs	0.82	0.68–1.00	0.054
Neoplasms	0.80	0.66–0.97	0.029
Diseases of the digestive system	0.76	0.62–0.92	0.007
Symptoms, signs, and ill-defined conditions	0.64	0.52–0.79	<0.001
Injury and poisoning	0.58	0.47–0.72	<0.001
Infectious and parasitic diseases	0.39	0.30–0.50	<0.001
Mental disorders	0.33	0.25–0.43	<0.001
Diseases of the nervous system and sense organs	0.32	0.24–0.41	<0.001
Diseases of the musculoskeletal system and connective tissue	0.16	0.11–0.22	<0.001
Complications of pregnancy, childbirth, and the puerperium	0.15	0.11–0.16	<0.001
Diseases of the skin and subcutaneous tissue	0.11	0.07–0.16	<0.001
Congenital anomalies	0.03	0.01–0.07	<0.001
Model 2. Incidence of mortality in age groups Log likelihood = −303.49, x^2^ = 1369.35 (3 df), *p* < 0.001	IRR	95% CI	*p*
Age group 30–59 years	0.85	0.77–0.95	0.005
Age group 60–79 years	1.37	1.28–1.47	<0.001
Age group ≥80 years	0.82	0.76–0.88	<0.001
Model 3. Mortality outcome Log likelihood = −8.55, x^2^ = 6.40 (1 df), *p* = 0.011	OR	95% CI	*p*
Comorbidities	1.36	1.01–1.81	0.038
Model 4. Mortality incidence by number of comorbidities Log likelihood = −55.90, x^2^ = 172.75 (5 df), *p* < 0.001	OR	95% CI	*p*
No comorbidities	1 ^a^		
One comorbidity	2.88	0.09–8.56	0.540
Two comorbidities	6.36	0.32–1.25	0.223
Three comorbidities	12.86	0.65–2.52	0.093
Four comorbidities	19.97	1.05–3.77	0.046
Five comorbidities	64.33	3.44–12.00	0.005

Note: IRR—Incidence Rate Ratio; OR—Odds Ratio; 95% CI—Confidence Interval; *p*—*p* value; a—reference category.

## Data Availability

The data included in this manuscript derived from the University database. We are not authorized to share the data with third-party organizations. However, the corresponding author is available to provide any explanation to the Editor if required.
